# Spatial Patterns of Global Peste Des Petits Ruminants Virus and Its Potential Risk Assessment for Various Wildlife Habitats

**DOI:** 10.1002/ece3.72723

**Published:** 2025-12-17

**Authors:** Guiping Lu, Feng Jiang, Rui Zhang, Jialong Guo, Lei Si, Wenrui Jiao, Daoxin Liu, Jingjie Zhang

**Affiliations:** ^1^ State Key Laboratory of Plateau Ecology and Agriculture Qinghai University Xining Qinghai China; ^2^ College of Agriculture and Animal Husbandry Qinghai University Xining China; ^3^ Key Laboratory of Adaptation and Evolution of Plateau Biota, Northwest Institute of Plateau Biology Chinese Academy of Sciences Xining Qinghai China; ^4^ College of Eco–Environmental Engineering Qinghai University Xining China

**Keywords:** climate and environmental variables, habitat, MaxEnt, PPRV, wildlife

## Abstract

The *Peste des Petits* Ruminants virus (PPRV) is highly contagious and capable of transmission through multiple host species. Wildlife, particularly endangered species, faces significant threats from this virus. In extreme scenarios, this may lead to the extinction of flagship and endangered species. This study integrated global PPRV epidemiological data (*n* = 902) and diverse environmental variables from 2014 to 2024, along with data from China spanning 2007–2024, to investigate the driving mechanisms of climatic and environmental factors on PPRV transmission and to assess the risk of PPRV infection in habitats suitable for wildlife. A PPRV cross‐species transmission risk assessment method based on MaxEnt has been developed. The study determined the importance of climatic and environmental parameters, including temperature seasonality, annual mean temperature, isothermality, mean diurnal range, mean monthly precipitation in March, and the maximum temperature of the warmest month, in influencing the spread of PPRV. On a global scale, the spread of PPRV exhibits distinct banded geographical distribution features, with the geographic location most likely occurring between 10°N and 50°N bands. The danger level progressively rises from west to east, with the distribution area primarily concentrated in a few Northern Hemisphere nations and regions. The potential threats to species such as *Camelus* within Camelidae, *Capra*, *Eudorcas*, and *Gazella* within wild Bovidae, and *Hydropotes* within Cervidae were a significant conservation concern. Our findings underscore the need for a cross‐border cooperative defense strategy to restore habitat connectivity and support the long‐term survival of these wildlife populations. This approach provides a scientific foundation for the protection of biodiversity and public health.

## Graphical abstract

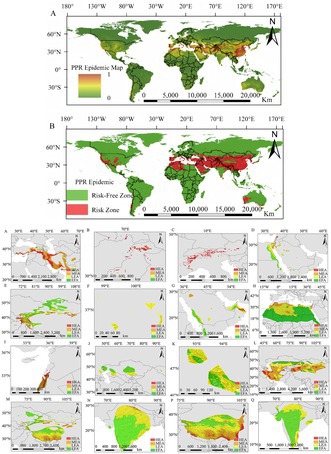

## 1 Introduction

Global biodiversity is under severe threat as a result of large‐scale alterations in the structure and function of the earth's ecosystem driven by human activities and global climate change (Kass 2020). According to the World Wide Fund for Nature's (WWF) *2024 Living Planet Report*, the average size of animal populations worldwide has shrank by almost 73%. A measure of this tendency is the Living Planet Index (LPI) (World Wide Fund for Nature (WWF), Zoological Society of London (ZSL) 2024), highlighting the extensive loss of biodiversity across the planet. Deforestation, environmental pollution, and climate change remain key drivers undermining the structure and stability of biodiversity (World Wide Fund for Nature (WWF), Zoological Society of London (ZSL) 2024), while infectious diseases are increasingly recognized as a significant factor threatening ecosystem integrity and species survival (Wiethoelter et al. 2015). Many species face an increasing risk of extinction in the next decades unless systematic, science‐based, and targeted conservation measures are implemented promptly. This would cause permanent deterioration and collapse of ecosystem functions (Kass 2020).

In the context of ongoing global biodiversity decline (World Wide Fund for Nature (WWF), Zoological Society of London (ZSL) 2024), wildlife infectious diseases have become a significant driver of species loss and ecosystem degradation (Gao et al. 2021). Peste des petits ruminants (PPR) is an acute and highly contagious viral disease caused by the small ruminant morbillivirus, a member of the Paramyxoviridae family (Xu et al. 2024). Peste des petits ruminants virus (PPRV) exhibits rapid transmission and high mortality rates, posing a significant threat to wild ruminant populations. The World Organization for Animal Health (OIE) and China have designated peste des petits ruminants virus (PPRV) as a Category A and Class I infectious disease, respectively, to facilitate targeted prevention and control measures (Xu et al. 2024; Pruvot et al. 2020). Research has shown that multiple outbreaks of Peste des petits ruminants (PPR) occurred in Mongolia between 2016 and 2017, resulting in the deaths of thousands of critically endangered Mongolian *Saiga tatarica* (*Saiga tatarica ssp. mongolica*) and triggering a severe conservation crisis (Pruvot et al. 2020). From 2020 to the present, data reported by the Animal Husbandry and Veterinary Bureau of the Ministry of Agriculture and Rural Affairs of the People's Republic of China, along with findings from related scientific literature, indicate that PPR has caused the deaths of more than 400 wild individuals (Kan et al. 2023; Bureau of Animal Husbandry and Veterinary Medicine, Ministry of Agriculture and Rural Affairs of the People's Republic of China 2020). According to data published by OIE, there have been 235 reported outbreaks of PPR worldwide, resulting in the deaths of over 100,000 ruminants, primarily sheep and goats (The World Organization for Animal Health (OIE) 2025). The pathogen of this outbreak was PPRV. It is traditionally believed that the virus primarily infects domestic goats and sheep. However, recent studies have confirmed that PPRV exhibits significant cross‐species transmission capabilities. Documented infections have been observed in at least 38 wildlife species (Li et al. 2017; Gür and Albayrak 2010; Balamurugan et al. 2012; SowjanyaKumari et al. 2021; Li et al. 2019), including the goitered gazelle ( *Gazella subgutturosa* ) (Gür and Albayrak 2010), Asiatic lion ( *Panthera leo persica* ) (Balamurugan et al. 2012), mountain gazelle ( *Gazella gazella* ) (SowjanyaKumari et al. 2021), Przewalski's gazelle ( *Procapra przewalskii* ) (Li et al. 2019), African savanna elephant ( *Loxodonta Africana* ) (SowjanyaKumari et al. 2021), and others. This study demonstrated that the transmission range of PPRV in wild animal populations exceeded expectations significantly, thereby posing a potential threat to numerous endangered and flagship species. It is imperative to enhance traditional research on its ecological transmission mechanism and protection impact. For example, in 2021, the infection by PPRV resulted in the death of a total of 371 individuals in the blue sheep ( *Pseudois nayaur* ) population (Fine et al. 2020). The data presented above indicate a gradual yet consistent expansion of PPRV from conventional cattle breeding systems to wild populations, alongside an accelerated trend of spillover events (Munir 2014). This dynamic markedly heightens the health risks encountered by wildlife, particularly endangered and flagship species, while also posing a significant challenge to the current prevention and control systems in place.

The conventional method for assessing the risk of PPRV primarily depends on epidemiological investigations and laboratory analyses. While it has made notable progress in the prevention and control of livestock diseases, there are significant limitations observed in the realm of wildlife protection: Firstly, obtaining samples from wild animals poses significant challenges, leading to a limited number of infected cases. This scarcity complicates the process of conducting large‐scale and systematic risk assessments (Jones et al. 2016). Secondly, traditional methods often struggle to adequately incorporate the ecological impacts of environmental factors such as altitude and temperature on virus transmission (Zhang et al. 2019). This limitation significantly constrains applicability in ecologically complex regions. The maximum entropy model (MaxEnt), serving as a species distribution model predicated on the probability density estimation of species occurrence data (Baluma Didier et al. 2024), offers an enhanced methodological framework for addressing such challenges. The model is capable of accurately predicting the potential distribution areas based on a range of environmental variables, even when the availability of species records is limited (Zhang et al. 2023). The field of infectious disease ecology, MaxEnt has been effectively utilized to analyze the risk of zoonotic transmission for the Ebola virus (EBoV) (Zhang et al. 2023), dengue virus (DENV) (Paz 2015), Zika virus (Cunze et al. 2019), and various other pathogens that have been predicted. These studies illustrate that the MaxEnt model demonstrates signifficant accuracy and reliability. According to the research findings, the MaxEnt model is remarkably accurate and dependable.

Therefore, utilizing the latest global distribution data on Peste des Petits Ruminants (PPR) epidemics and integrating high‐resolution environmental variables, this study developed a MaxEnt model informed by crucial climatic factors. The objective is to systematically assess the mechanisms driving the influence of various environmental factors on PPRV transmission and to evaluate the risk of PPRV infection in wild animals that are suitable for habitation. Through comprehensive model analysis, this study aims to address the following fundamental scientific problems: how environmental factors affect the potential distribution of PPRV. How to use the MaxEnt model to analyze the spatial risk distribution characteristics of global PPRV in the context of limited monitoring data. The distribution pattern and difference of PPRV risk in different types of wild animal distribution areas were evaluated. The results will provide theoretical support for understanding the ecological transmission mechanism of PPRV in different environmental areas and also provide a key scientific basis for disease prevention and control of endangered wild animals and early warning of cross‐species transmission, which has important ecological protection and disease management significance.

## 2 Materials and Methods

### 2.1 Collection and Statistical Analysis of Distribution Sites of Peste Des Petits Ruminants Epidemic Situation

The geographical distribution data of PPR used in this study were primarily sourced from multiple authoritative databases and official documents. Data on PPR cases globally from 2014 to 2024 were mainly obtained from the OIE database, while case data from within China for the years 2007–2014 were derived from relevant literature and reports. To ensure the timeliness and completeness of the data, the study further collected PPR outbreak information recorded in the “Veterinary Bulletin” published by the Animal Husbandry and Veterinary Bureau of the Ministry of Agriculture and Rural Affairs of the People's Republic of China from 2015 to 2024. The total number of recorded PPR outbreaks amounts to 981. Additionally, for each viral record, relevant attribute information was gathered, including the location of occurrence (province, city, and latitude/longitude), reporting date, host type, number of cases, and number of fatalities, among other core parameters.

Based on real‐world conditions, the PPR geographical distribution data utilized in this study encompasses epidemiological information derived from both wildlife and domestic animals. Given that the activity ranges of domestic animals significantly overlap with the habitats of wildlife, existing research has confirmed that outbreaks in domestic animals can impact the risk levels for wildlife (Aziz et al. 2016). Therefore, this study integrates epidemiological data from both wildlife and domesticated animals to provide a more comprehensive epidemiological context, thereby enhancing the model's accuracy.

ArcGIS 10.8 software was used to filter the epidemic's spatial distribution locations in order to reduce the divergence brought on by spatial autocorrelation between samples. To maximize the spatial independence of the data, the minimum spatial resolution distance was established at 1 km in order to eliminate duplicate or excessively adjacent sample records. A total of 902 PPRV outbreak records were retained after screening. Sorting and storing the selected epidemic distribution data in a ‘.csv’ file in the ‘species‐longitude‐latitude’ format allowed for additional study and modeling. This process provides a robust database for the further development of the MaxEnt model and ensures the geographic precision of the input data and the scientific nature of the modeling.

### 2.2 Environmental Data Collection and Processing

This study selected 68 bioclimatic variables that were highly correlated with the geographic distribution of PPRV from the Global Climate Data (http://www.worldclim.org/) (Table 1). These variables were calculated based on climate elements such as precipitation and temperature through a series of climate modeling functions and are widely used in biogeographic modeling, effectively representing the main environmental factors that limit the spatial distribution of PPRV. They were often utilized in biogeographic modeling and possessed the ability to accurately delineate the primary environmental constraints that limited the regional distribution of PPRV. The spatial resolution of the chosen climate variable data was 30 arc‐seconds (around 1 km), which satisfied the accuracy criteria of species distribution modeling at the regional scale. ArcGIS 10.8 was utilized to change the format of all climate variable data in order to implement the next application in the MaxEnt model, and the unified output was ‘.asc’ grid format. The WorldCom 1.4 version selected for this study was the average data spanning 50 years, from 1950 to 2000.

**TABLE 1:** The name and description of WorldClim data.

Code	Data description
Bio1	Annual mean temperature
Bio2	Mean diurnal range
Bio3	Isothermality (Bio2/Bio7) (×100)
Bio4	Temperature seasonality (standard deviation × 100)
Bio5	Max temperature of warmest month
Bio6	Min temperature of coldest month
Bio7	Temperature annual range (Bio5–Bio6)
Bio8	Mean temperature of wettest quarter
Bio9	Mean temperature of driest quarter
Bio10	Mean temperature of warmest month
Bio11	Mean temperature of coldest month
Bio12	Annual precipitation
Bio13	Precipitation of wettest month
Bio14	Precipitation of driest month
Bio15	Precipitation seasonality (coefficient of variation)
Bio16	Precipitation of wettest quarter
Bio17	Precipitation of driest quarter
Bio18	Mean temperature of warmest quarter
Bio19	Mean temperature of coldest quarter
Tmean1–12	Average monthly mean temperature
Tmin1–12	Average monthly minimum temperature
Tmax1–12	Average monthly maximum temperature
Prec1–12	Average monthly precipitation
Alt	Altitude (elevation above sea level)

This study first extracted the environmental variable attribute values corresponding to the distribution point of PPRV cases using ArcGIS 10.8 software. This was done in order to decrease the information redundancy between bioclimatic variables and enhance the stability and explanatory power of model prediction. Second, the correlation between the retrieved variables was examined using the SPSS 22 program. The Pearson correlation coefficient is utilized for quantitative assessment because the environmental variables that were chosen are continuous variables. The two variables are deemed to be highly connected if the correlation coefficient's absolute value is more than 0.8 (Dormann Carsten et al. 2013). Consequently, variables that exhibited low correlation yet held significant ecological importance were prioritized for the MaxEnt model generation, while those with high redundancy and relatively weak ecological interpretation were excluded.

### 2.3 Construction of Global Peste Des Petits Ruminants Risk Model

The MaxEnt 3.3.3 k model was used in this investigation. To find the best parameter combination, many parameters were set in the feature class selection and regularization multiplier (*β*) before building the risk model. In feature class selection, various *β* values were set from 1 to 5 every 0.5 in the “Regularization multiplier,” and Linear, Quadratic, Product, Threshold, and Hinge were combined, respectively. The model operation results under each set of parameters were entered into ENMTools in the “points file, model asc file, model lambdas file” format, and the AICc and BIC values were computed for each set of parameters. The parameters with the lowest AICc and BIC scores were utilized as the ideal parameters for building the virus risk model for PPR (Table 2), in conjunction with the response curve's smoothness under various parameters (smoothness can be understood biologically). The distribution site data of PPR were randomly split into two groups of data after the ideal parameters were chosen. Of these, the risk model was built using 75% of the distribution data, and the accuracy of the model was checked using the remaining 25% of the distribution site data. Simultaneously, the jackknife approach was chosen to assess the rate of environmental elements contribution, and it was repeated ten times. It chooses the output file in the ‘.asc’ format. Additionally, to assess spatial independence, we measured, for 10 independent random splits, the distance from each test occurrence to its nearest training occurrence. In split *i*, we took the test set T*i* from the Samples with data table (Test or train = Test) and defined the training set R*i* (all occurrences Oexcluding T*i*) to avoid overlap. Coordinates (WGS84 lon/lat) were cleaned by removing missing values, deduplicating identical coordinates, and standardizing decimal precision. For each point in T*i*, we computed great‐circle (Haversine) distances to all points in R*i* (Earth radius 6371 km) and recorded the minimum as its nearest‐neighbor distance. For each split, we summarized min, P25, median, P75, max; pooling all per‐point distances from the 10 splits, we also reported the overall median and interquartile range (P25–P75).

**TABLE 2:** AICc and BIC score table under different parameters of MaxEnt model. Bolded entries indicate the optimal parameter model.

Serial number	Feature class selection	Regularization multiplier (*β*)	Parameters	InL	BIC score	AICc score
1	L, Q	1	24	−16135.86	32435.02	32321.08
2	L, Q	1.5	24	−16135.86	32435.02	32321.08
3	L, Q	2.0	24	−16135.86	32435.02	32321.08
4	L, Q	2.5	25	−16134.45	32439.02	32321.08
5	L, Q	3.0	22	−16138.77	32427.24	32320.39
6	L, Q	3.5	23	−16141.67	32439.85	32322.69
7	L, Q	4.0	22	−16142.01	32433.73	32330.60
8	**L, Q, P**	**1**	**52**	**−15977.62**	**32309.09**	**32329.18**
9	L, Q, P	1.5	52	−15977.62	32309.09	32065.74
10	L, Q, P	2.0	52	−15977.62	32309.09	32065.74
11	L, Q, P	2.5	53	−15981.18	32323.01	32065.74
12	L, Q, P	3.0	54	−15985.37	32338.19	32065.74
13	L, Q, P	3.5	52	−16005.44	32364.73	32075.11
14	L, Q, P	4.0	50	−15995.64	32331.51	32085.75
15	L, Q, P, H	1	115	−15719.02	32220.56	32121.38
16	L, Q, P, H	1.5	112	−15786.05	32334.22	32097.27
17	L, Q, P, H	2.0	100	−15826.15	32332.76	31748.86
18	L, Q, P, H	2.5	93	−15860.49	32353.81	31701.98
19	L, Q, P, H	3.0	92	−15905.90	32437.83	31828.19
20	L, Q, P, H	3.5	78	−15931.74	32394.24	31877.52
21	L, Q, P, H	4.0	72	−15946.29	32382.52	32049.27

In this study, the accuracy of the risk model was also assessed using the receiver operating characteristic curve (ROC curve) and the area under the ROC curve (AUC value). AUC values varied between 0.5 and 1. The model's accuracy and dependability increased with its AUC value. Each individual environmental variable was generated under conditions consistent with the main model, including occurrence points, background samples, data partitioning, and parameter settings. The AUC value corresponding to each individual variable was used to measure the amount of species distribution information it independently conveys, with higher values indicating stronger independent explanatory power. All evaluations were conducted under the same repeated partitions (10 iterations), and we reported the mean AUC ± standard deviation for each variable. We established approximately 10,800 background points, a number chosen to adequately represent the environmental context of the study area while maintaining computational efficiency. These background points were generated using simple random sampling within the geographical boundaries of the entire study area. To ensure the robustness of the model evaluation process, we conducted ten repeated runs, with background points in each run being independently generated according to the previously outlined rules. The model produced highly consistent output results across all repetitions.

ArcGIS 10.8 software was used to import the outcomes of MaxEnt's simulation of the risk distribution of PPR. The habitat range of wild species may be downloaded by clicking on the download page selection (Range data—Points (CSV)) once the correct species has been queried on the IUCN Red List of Threatened Species' official website. Get the Global National Regions from the Nature Earth website (https://www.naturalearthdata.com/) and import the ArcGIS 10.8 program. The following terms were highlighted in blue once you entered the download interface: GeoPackage (436 MB), SHP (576 MB), SQLite (423 MB), and Natural Earth Quick Start Kit (219 MB). It was possible to use ArcGIS 10.8 as the base layer after importing the software. The decompression was downloaded after selecting SHP.

In this study, the classification was based on the mean logistic threshold of Maximum Training Sensitivity Plus Specificity, with a mean value of 0.225. At this threshold, the information missing rate in the test set was 0.0727 ± 0.0203, compared to 0.0615 ± 0.0056 in the training set, which corresponded to a predicted area of 0.2227 ± 0.0062. Additionally, the model's Test AUC was 0.9064 ± 0.0128. These results were based on 10 repetitions to ensure both reproducibility and robustness. In this study, the threshold calculated by the MaxEnt model was 0.225. Therefore, areas with a threshold of ≥ 0.225 were classified as risk areas, while areas with a threshold of < 0.225 were classified as risk‐free areas (extremely low‐risk areas). This threshold (0.225) is closely aligned with the commonly used risk probability threshold (0.2) in the ArcGIS reclassification function. To facilitate spatial analysis and result presentation, this study subsequently adopted the ArcGIS reclassification standard for risk zone division:risk probability ≥ 0.6 was classified as a high‐risk area, 0.4–0.6 as a medium‐risk area, 0.2–0.4 as a low‐risk area, and < 0.2 as a very low‐risk or risk‐free area (Zhang et al. 2019). Therefore, the subsequent research was divided and analyzed according to the ArcGIS reclassification function.

## 3 Results

### 3.1 Parameter Optimization Analysis and Verification of the Global Peste Des Petits Ruminants Model

After removing the variables with a correlation coefficient higher than 0.8, 16 environmental variables with low correlation and higher biological significance were chosen for MaxEnt model analysis based on the Pearson correlation coefficient calculation of 68 environmental variables. These variables included temperature seasonality (standard deviation × 100) (Bio4), annual mean temperature (Bio1), isothermality (Bio3), mean diurnal range (Bio2), altitude (Alt), and mean monthly precipitation in March (Prec3), maximum temperature of warmest month (Bio5), precipitation of driest month (Bio14), minimum temperature of coldest month (Bio6), the seasonality of precipitation (coefficient of variation) (Bio15), mean temperature of warmest quarter (Bio18), mean temperature of coldest quarter (Bio19), mean temperature of wettest quarter (Bio8), mean monthly precipitation in July (Prec7), mean monthly precipitation in January (Prec1), and annual precipitation (Bio12). The chosen environmental variables were separated into three categories: variables relating to temperature, rainfall, and terrain.

A global risk model of PPRV was constructed in this work using 21 sets of distinct parameter combinations, and the AICc and BIC values of each model under various parameter settings were computed. According to the analysis, the seven parameter combinations with the L + Q + *P* + T combination had relatively low AICc and BIC scores, and the response curves under each parameter were not smooth. As a result, these combinations were not chosen. The seven parameter sets of the L + Q combination and the seven parameter sets of the L + Q combination, on the other hand, exhibited a smooth response region. The combination L + Q + *P* with *β* = 1.0 was selected as the optimal model parameter (Table 2) due to its lowest AICc and BIC scores among the 14 parameter combinations, along with a generally flat response curve. Ten repeat operations were then set. Under 10 independent random splits, the distance distribution from test presences to their nearest training presences showed clear spatial separation: median 29.65 km, P25–P75 = 12.49–53.07 km. Most test samples were substantially separated from training samples, indicating a low risk of near‐neighbor information leakage. The distributional shapes were consistent across splits, and spatial separation remained stable (accessory Table S1 and Figure S1). Using 10 calculations, the MaxEnt model's ROC curve was produced. AUC_test_, or average area under the ROC curve, was 0.906 ± 0.012, exceeding the threshold of 0.9. These results demonstrate excellent and highly credible predictive performance, supporting the model's use in subsequent analysis.

### 3.2 Analysis of the Coupling Relationship Between the Spatial Distribution of Peste Des Petits Ruminants and Various Environmental Variables

The results showed that among the selected 16 environmental variables, temperature seasonality (standard deviation × 100) (Bio4, 30.561%), annual mean temperature (Bio1, 21.693%), isothermality (Bio2/Bio7 × 100) (Bio3, 9.508%), mean diurnal range (Bio2, 9.157%), altitude (alt, 5.760%), mean monthly precipitation in March (Prec3, 5.616%), and maximum temperature of warmest month (Bio 5, 5.213%) had higher contribution rates to PPRV. Each of these variables had a single contribution rate greater than 5%, making them the main environmental factors affecting the potential risk distribution of PPRV (Table 3). It had an overall contribution rate of 87.508%. These three groups of factors—temperature, precipitation, and altitude—were among the seven environmental variables with the highest contribution rates. The contribution rates of precipitation of the driest month (Bio14), minimum temperature of the coldest month (Bio6), precipitation coefficient of variation (Bio15), mean temperature of warmest quarter (Bio18), mean temperature of the coldest quarter (Bio19), mean temperature of the wettest quarter (Bio8), mean monthly precipitation in July (Prec7), January mean monthly precipitation (Prec1), and annual precipitation (Bio12) to the potential risk of PPRV were all less than 5% (Table 3).

**TABLE 3:** Analysis of the relative contribution rate and AUC value of each climate environment variable factor in MaxEnt model.

Single‐variable	Contribution percentage (%)	Permutation importance (%)	Gain value	AUC
Bio4	30.561 ± 0.650	7.083 ± 1.125	0.385 ± 0.012	0.766 ± 0.028
Bio1	21.693 ± 0.608	36.089 ± 1.706	0.577 ± 0.007	0.797 ± 0.016
Bio3	9.508 ± 1.426	13.454 ± 2.758	0.414 ± 0.019	0.798 ± 0.028
Bio2	9.157 ± 1.136	2.500 ± 0.402	0.016 ± 0.001	0.553 ± 0.023
Alt	5.760 ± 0.491	5.922 ± 0.449	0.050 ± 0.004	0.553 ± 0.032
Prec3	5.616 ± 0.581	1.341 ± 0.262	0.134 ± 0.004	0.667 ± 0.019
Bio5	5.213 ± 0.531	0.754 ± 0.433	0.206 ± 0.005	0.663 ± 0.018
Bio14	3.897 ± 0.392	4.992 ± 0.539	0.068 ± 0.002	0.587 ± 0.018
Bio6	3.835 ± 1.227	8.396 ± 1.450	0.267 ± 0.004	0.694 ± 0.020
Bio15	1.182 ± 0.459	0.992 ± 0.256	0.131 ± 0.004	0.662 ± 0.026
Bio18	0.921 ± 0.235	1.785 ± 0.493	0.013 ± 0.010	0.567 ± 0.037
Bio19	0.818 ± 0.351	7.692 ± 1.169	0.014 ± 0.005	0.635 ± 0.020
Bio8	0.636 ± 0.377	1.348 ± 0.801	0.015 ± 0.001	0.521 ± 0.032
Prec7	0.437 ± 0.176	1.346 ± 0.498	0.041 ± 0.002	0.547 ± 0.042
Prec1	0.396 ± 0.179	4.474 ± 1.404	0.059 ± 0.003	0.634 ± 0.026
Bio12	0.370 ± 0.244	1.831 ± 0.656	0.022 ± 0.003	0.591 ± 0.025

The greatest gain value generated by the jackknife method was analyzed and calculated. The gain value test results in Table 2 showed the importance of each component. Temperature seasonality (standard deviation × 100) (Bio4), annual mean temperature (Bio1), isothermality (Bio3), mean diurnal range (Bio2), altitude (Alt), mean monthly precipitation in March (Prec3), and maximum temperature of the warmest month (Bio5) were all significant environmental factors for the PPRV risk distribution model's construction, according to the table values.

The study evaluated the relative importance of each environmental variable based on MaxEnt's jackknife method. Specifically, separate models were built for each variable (only‐variable models), while maintaining consistency in occurrence points, background samples, and data partitions. The ROC‐AUC for the test set was then calculated, and the results were compared with models that excluded the variables (without‐variable models) and those that included all variables (with‐all models). In the single‐variable models, the AUC of each variable reflected the accuracy of its individual model, indicating that a higher prediction accuracy for a certain environmental factor corresponded to a lower predictive accuracy for others, and vice versa. Isolating the variables allowed for a deeper understanding of their independent explanatory power for the distribution patterns. Higher AUC values indicated that the variable itself produced a strong discriminatory effect. In this study, the AUC values for the temperature seasonal variation coefficient (standard deviation × 100) (Bio4), the annual average temperature (Bio1), and isotherm (Bio3) ranged from 0.7 to 0.8, indicating good accuracy. The AUC values for March precipitation (Prec3), the highest temperature in the warmest month (Bio5), the lowest temperature in the coldest month (Bio6), precipitation coefficient of variation (Bio15), precipitation in the coldest quarter (Bio19), and January precipitation (Prec1) ranged from 0.6 to 0.7, indicating average accuracy. AUC values for the remaining climate and environmental factors were all below 0.6, suggesting that these factors had relatively little influence on the construction of the PPRV risk distribution model (Table 3).

**FIGURE 1:**
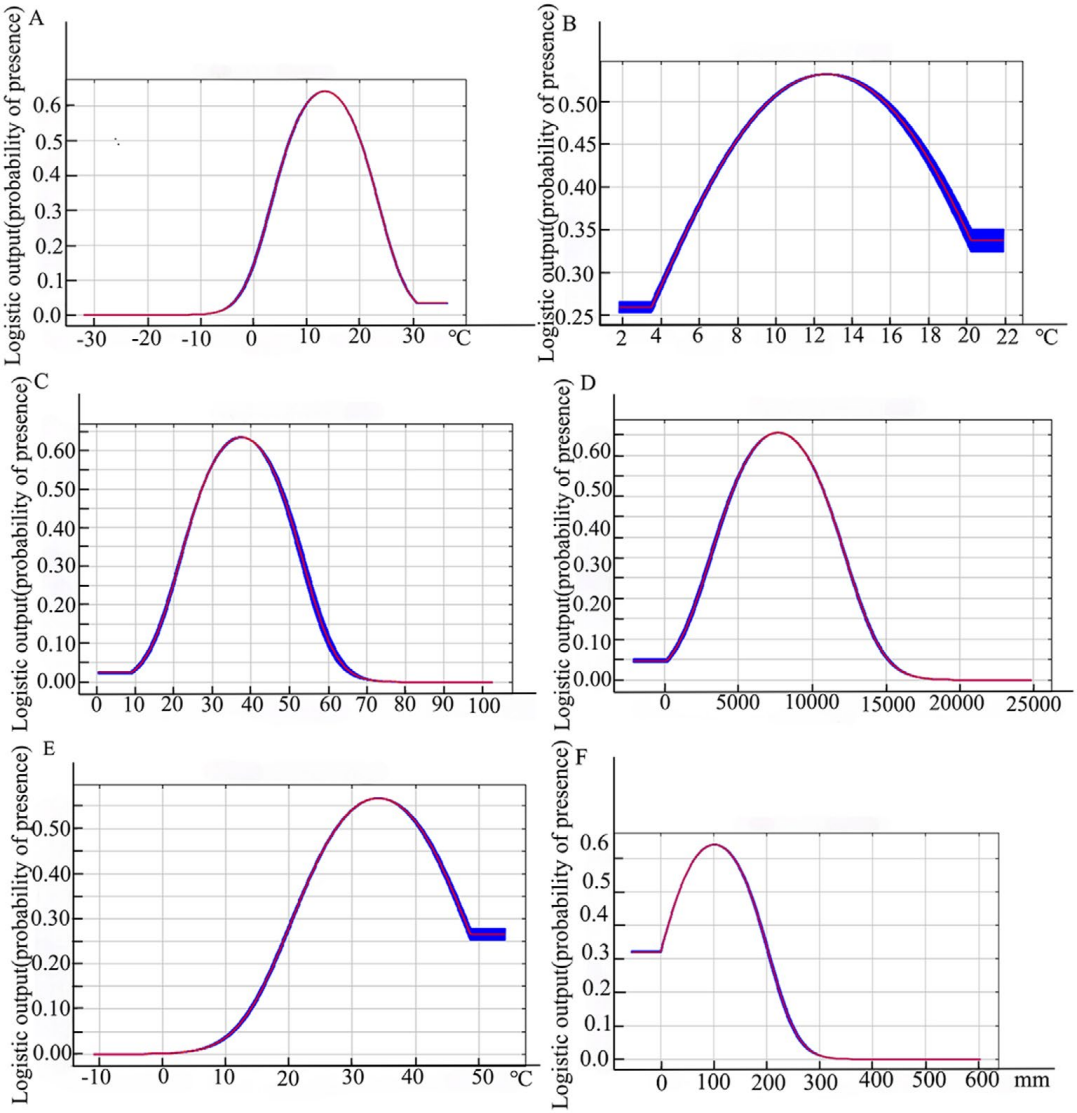
The response curve of the relationship between climate environment variables and PPRV with different contribution rates. MaxEnt predicted the response curve of the effect of a single climate variable on PPRV. (A) Annual Mean Temperature (Bio1). (B) Mean Diurnal Range (Bio2). (C) Isothermality (Bio2/Bio7) (×100) (Bio3). (D) Temperature seasonality (standard deviation × 100) (Bio4). (E) Max Temperature of Warmest Month (Bio5). (F) Mean monthly precipitation (Prec3).

In the collected data of 902 cases, the annual mean temperature (Bio1) ranged from −7°C~28°C, and the temperature seasonality (standard deviation × 100) (Bio4) ranged from 20~1620. The annual mean temperature was between −7°C and 18°C, and the temperature seasonality was between 20 and 880. With the increase of annual mean temperature and seasonal the seasonal variation coefficient of temperature, the risk probability became greater, with the annual mean temperature value reaching 17.5°C and the temperature seasonality at 880. As the seasonal variation coefficient of annual mean temperature and temperature seasonality decreased, the risk of PPRV gradually diminished (Figure 1A,D). Isothermality (Bio3) and mean diurnal range (Bio2) reflected the temperature difference between day and night and seasonal temperature difference, respectively. The optimum values were 3.5°C and 12.25°C (Figure 1B,C), indicating that a medium temperature difference environment was conducive to the survival of PPRV.

Rainfall and high temperatures had an impact on PPRV, as shown by the March mean monthly precipitation (Prec3) and the warmest month's maximum temperature (Bio5), respectively (Figure 1E,F). The findings indicated that the strongest conditions for the virus's survival were a maximum temperature of roughly 32°C during the warmest month and precipitation between 0 and 100 mm. When these peaks were surpassed, the March mean monthly precipitation and the warmest month's maximum temperature were negatively correlated with the PPRV.

### 3.3 Global Peste Des Petits Ruminants Risk Distribution Pattern and Area Analysis of Each Risk Level

**FIGURE 2:**
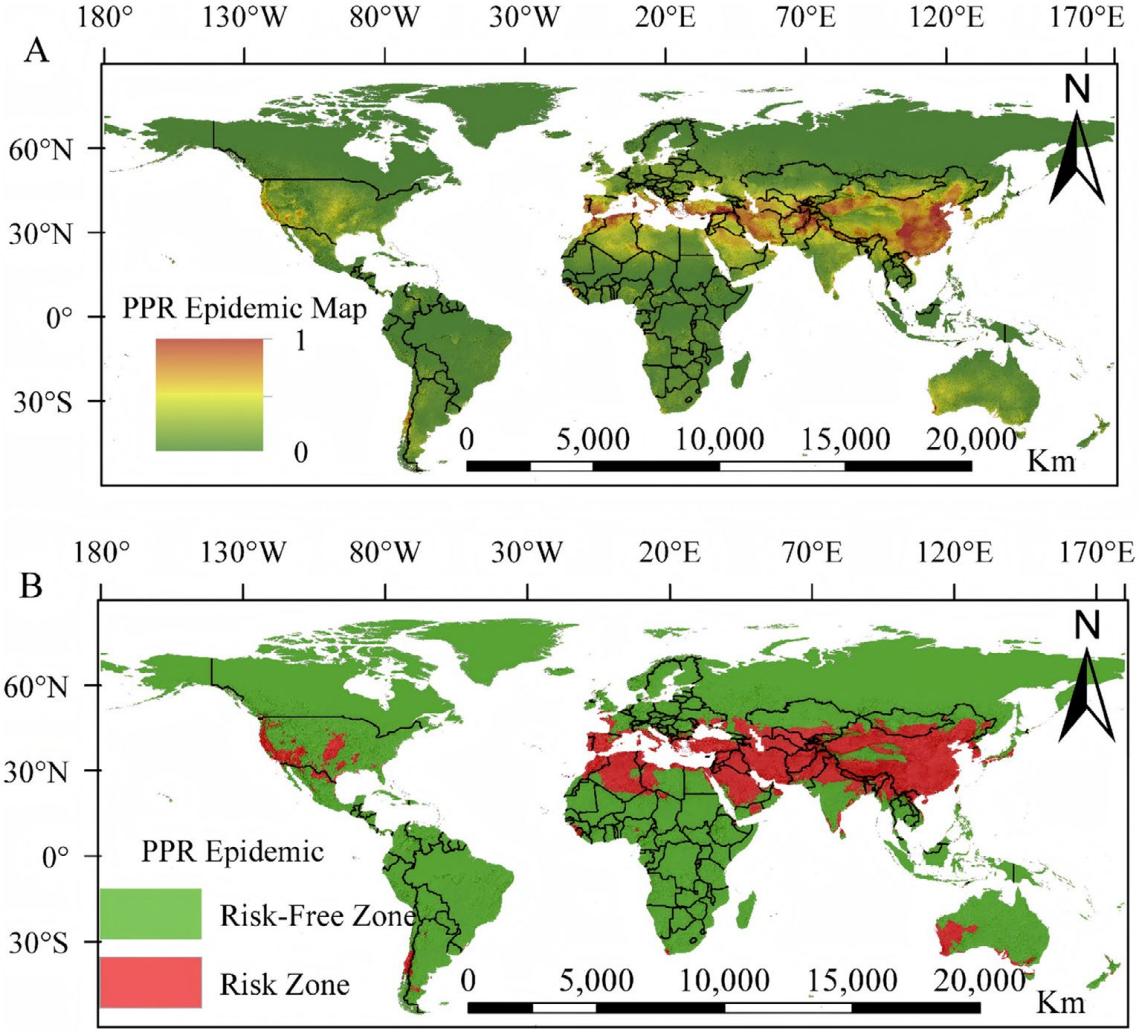
Global PPRV risk distribution map. (A) Red indicates risk zone area, Green indicates risk‐free zone area. (B) Red and other colors indicates risk zone area. Green indicates risk‐free zone area.

The high‐risk areas of PPRV were predominantly located in Asia, Europe, and Africa in the northern hemisphere. From west to east, the danger of PPR increased gradually and appeared as a band. The geographical position was roughly a banded range from 10°N~50°N. Additionally, the Southern Hemisphere had a comparatively small number of high‐risk regions, primarily located in northern Australia and southern Africa (Figure 2B). In particular, the majority of Asia's high‐risk regions were located in China, southern Mongolia, western Kyrgyzstan, eastern Tajikistan, southern Kazakhstan, the southern Turkish Mediterranean coast, among other countries. In Europe, the primary locations of PPRV high‐risk areas were along the Black Sea coasts of southern Greece, southwestern Romania, and the Mediterranean coast of southwestern Albania. The Mediterranean coast of northern Algeria, thecoast of the Mandeb Strait in western Djibouti, and the Gibraltar Strait in northwest Morocco were among the countries in the African region that posed the highest risk of PPRV. The majority of PPRV cases in the Americas were found in western Canada. By employing thresholds derived from the model's operational conditions, we determined the average of 10 results (0.225) as the basis for threshold classification. The results indicated that the area classified as a danger zone was 18,357,631 km^2^.

### 3.4 Overall Analysis of Peste Des Petits Ruminants Risk in Different Wildlife Distribution Areas

**FIGURE 3:**
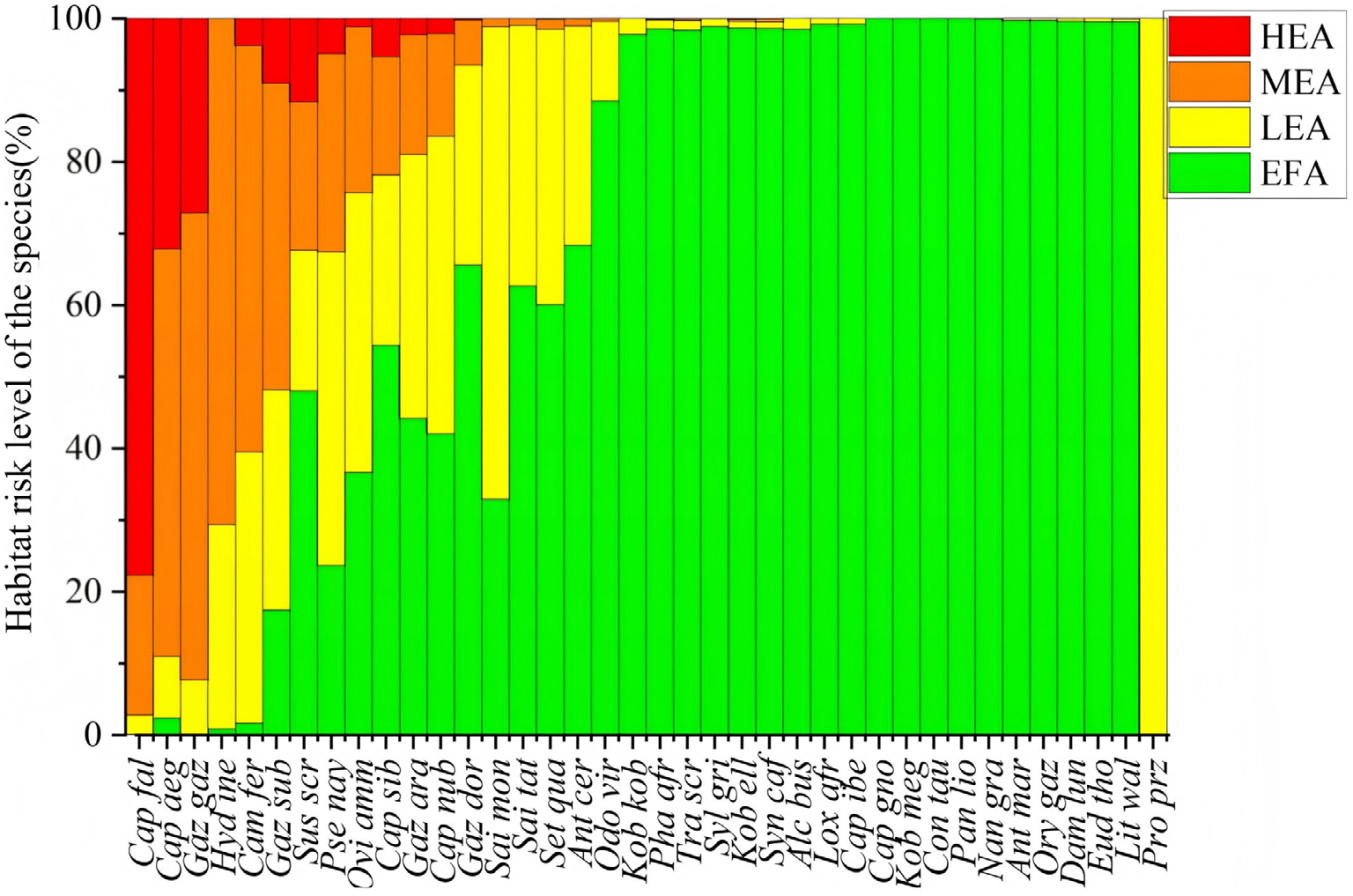
The distribution ratio of species habitat to disease risk areas. Red indicates high‐risk areas, orange indicates medium‐risk areas, yellow indicates low‐risk areas, and green indicates very low‐risk (risk‐free) areas.

This result facilitated subsequent research. The study included a total of 902 instances among which 38 wild animal species were found to be susceptible to PPRV infection (Table 4). It constituted a significant potential threat to multiple species, including Bovidae (*N* = 31), Cervidae (*N* = 2), Elephantidae (*N* = 1), Felidae (*N* = 1), Camelidae (*N* = 1), Suidae (*N* = 2), and wild animals from other families. According to this study, the level of threat that various species experienced varied significantly (Figure 3). For example, PPRV accounted for about 75% of the high‐risk zones and posed the highest risk to markhor ( *Capra falconeri* ). Additionally, over 90% of the high‐risk areas were inhabited by mountain gazelles ( *Gazella gazella* ) and wild goats ( *Capra aegagrus* ), with more than 25% of these habitats classified as high‐risk. In contrast, less than 20% of the high‐risk areas were associated with species such as goitered gazelle ( *Gazella subgutturosa* ), wild camel ( *Camelus ferus* ), and water deer ( *Hydropotes inermis* ), which together constituted more than 50% of the high‐risk areas. Conversely, Arabian gazelle ( *Gazella arabica* ) and African wild boar ( *Sus scrofa* ) were less affected, constituting less than 10% of the total and were primarily found in medium‐ and low‐risk locations.

**TABLE 4:** Wild hosts infected with PPRV.

Family	Genus	Latin name	Scientific name	Endangered grade
Bovidae	Capra	*Capra falconeri*	Markhor	NT, 2015
	Capra	*Capra ibex*	Alpine Ibex	LC, 2023
	Capra	*Capra nubiana*	Nubian Ibex	VU, 2020
	Capra	*Capra sibirica*	Siberian Ibex	NT, 2020
	Capra	*Capra aegagrus*	Wild Goat	NT, 2020
	Gazella	*Gazella subgutturosa*	Goitered Gazelle	VU, 2016
	Gazella	*Gazella arabica*	Arabian Gazelle	VU, 2016
	Gazella	*Gazella dorcas*	Dorcas Gazelle	VU, 2016
	Gazella	*Gazella gazella*	Mountain Gazelle	EN, 2016
	Kobus	*Kobus ellipsiprymnus*	Waterbuck	LC, 2016
	Kobus	*Kobus kob*	Kob	LC, 2016
	Kobus	*Kobus megaceros*	Nile Lechwe	EN, 2016
	Connochaetes	*Connochaetes gnou*	Black Wildebeest	LC, 2016
	Connochaetes	*Connochaetes taurinus*	Common Wildebeests	LC, 2016
	Saiga	*Saiga tatarica*	Saiga	NT, 2023
	Saiga	*Saiga tatarica ssp. Mongolica*	Mongolian Saiga	EN, 2018
	Eudorcas	*Eudorcas thomsonii*	Thomson's Gazelle	LC, 2018
	Sylvicapra	*Sylvicapra grimmia*	Common Duiker	LC, 2016
	Litocranius	*Litocranius walleri*	Gerenuk	NT, 2016
	Oryx	*Oryx gazella*	Gemsbok	LC, 2016
	Ovis	*Ovis ammon*	Argali	NT, 2020
	Nanger	*Nanger granti*	Grant's Gazelle	LC, 2016
	Antelope	*Antilope cervicapra*	Blackbuck	LC, 2016
	Antidorcas	*Antidorcas marsupialis*	Springbok	LC, 2016
	Alcelaphus	*Alcelaphus buselaphus*	Hartebeest	LC, 2015
	Damaliscus	*Damaliscus lunatus*	Topi	LC, 2016
	Syncerus	*Syncerus caffer*	African Buffalo	NT, 2018
	Tetracerus	*Tetracerus quadricornis*	Four‐horned Antelope	VU, 2016
	Tragelaphus	*Tragelaphus scriptus*	Bushbuck	LC, 2016
	Procapra	*Procapra przewalskii*	Przewalski's Gazelle	EN, 2016
	Pseudois	*Pseudois nayaur*	Blue Sheep	LC, 2014
Elephantidae	Loxodonta	*Loxodonta africana*	African Savanna Elephant	EN, 2020
Camelidae	Camelus	*Camelus Ferus*	Wild Camel	CR, 2008
Cervidae	Odocoileus	*Odocoileus virginianus*	White‐tailed Deer	LC, 2015
	Hydropotes	*Hydropotes inermis*	Water Deer	VU, 2014
Felidae	Panthera	*Panthera leo ssp. Persica*	Asiatic Lion	EN, 2023
Suidae	Phacochoerus	*Phacochoerus africanus*	Common Warthog	LC, 2016
	Sus	*Sus scrofa*	Wild Boar	LC, 2018

PPRV's habitat distribution had a direct impact on its dangerous wildlife. The analysis revealed that variations in temperature and precipitation had a significant effect on PPRV's geographic distribution. The risk of PPRV transmission was highest at an annual mean temperature of 17.5°C, while the likelihood of virus transmission peaked at a temperature seasonality of approximately 880. The aforementioned findings suggested that certain climatic conditions had a significant impact on PPRV transmission. Particularly in tropical and subtropical regions, the potential distribution of PPRV may have increased as global climate change intensified. Together, these environmental variables greatly raised the likelihood of PPRV transmission in specific areas, jeopardizing the survival of wild animals.

### 3.5 Identification and Prediction of PPRV Potential Risk Areas in Wild Animal Habitats of Bovinae

**FIGURE 4:**
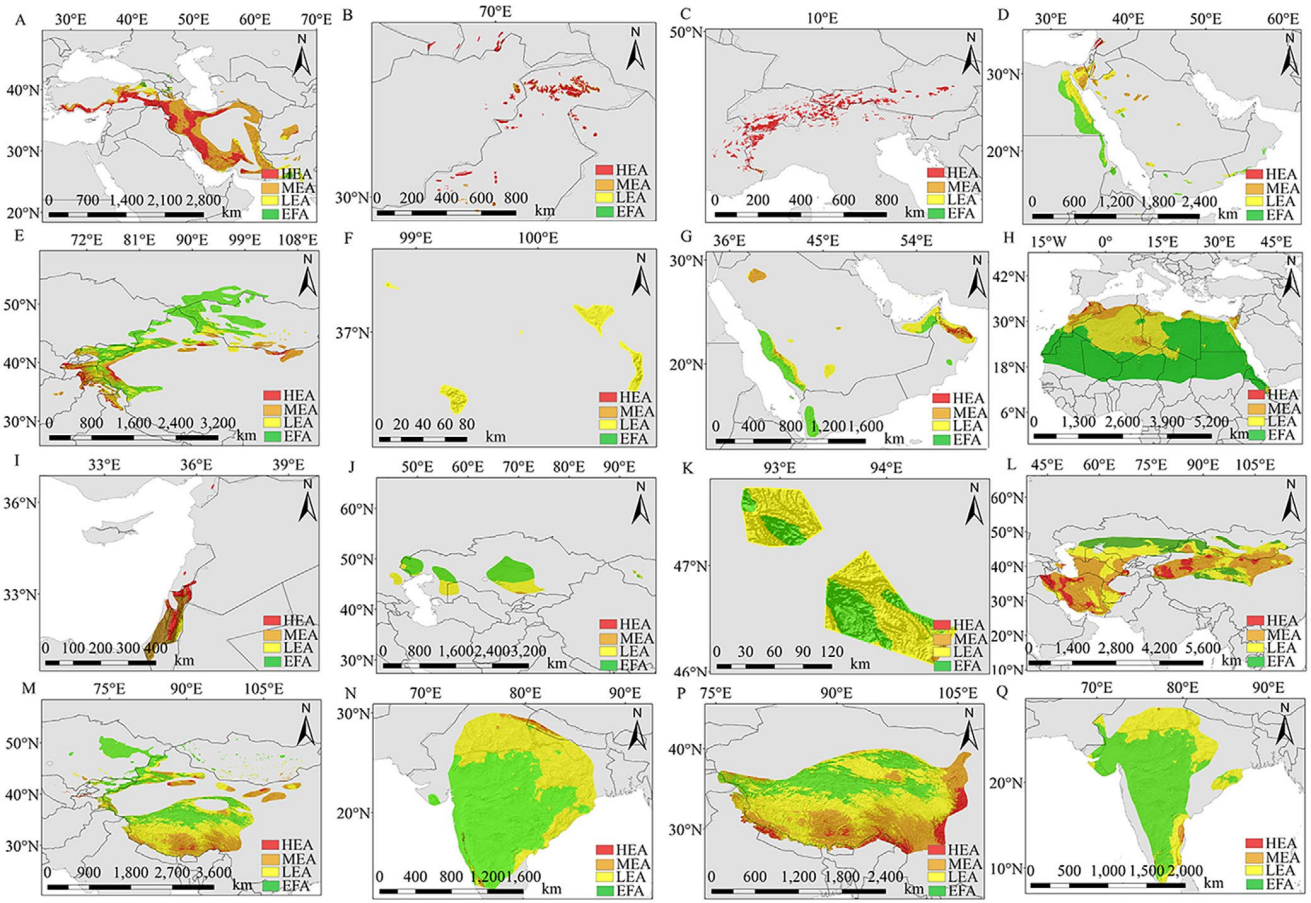
Identification and analysis of potential high‐risk areas of peste des petits ruminants in wild animal distribution areas of Bovinae. (A) Wild Goat; (B) Markhor; (C) Alpine Ibex; (D) Nubian Ibex; (E) Siberian Ibex; (F) Przewalski's Gazelle; (G) Arabian Gazelle; (H) Dorcas Gazelle; (I) Mountain Gazelle; (J) Saiga; (K) Mongolian Saiga; (L) Goitered Gazelle; (M) Argali; (N) Four‐horned Antelope; (P) Blue Sheep; (Q) Blackbuck.

This study involved a total of 38 wild animal species, including 31 species of the Bovidae family, among which 16 species were distributed in high‐risk areas for PPRV potential distribution (Figure 3). The study noted that all species found in high‐risk areas were located between 20°N and 70°N (Figure 4), with 10 species distributed in mid‐to‐high latitude regions (defined as being between 30° and 90°). The habitats of these species were predominantly located at altitudes above 1500 m. In these mid‐to‐high latitude regions, the threat posed by PPRV to wildlife species was higher than in low‐latitude areas.

According to the analysis of Figure 4, different wild animals such as wild goats ( *Capra aegagrus* ), markhor ( *Capra falconeri* ), alpine ibex ( *Capra ibex* ), Przewalski's gazelle ( *Procapra przewalskii* ), mountain gazelle ( *Gazella gazella* ), Siberian ibex ( *Capra sibirica* ), saiga ( *Saiga tatarica* ), Mongolian saiga (*Saiga tatarica ssp. mongolica*), argali ( *Ovis ammon* ), goitered gazelle ( *Gazella subgutturosa* ), and blue sheep ( *Pseudois nayaur* ) were active in the range of 30°N~50°N. Most species were active at the middle or high latitudes, while some could be found across all three. The distribution of these species' habitats was examined, revealing that they were present between 0 and 5500 m. However, the species found above 1500 m were considered more significant than those located at that elevation. The range of activities for these species was primarily characterized by a temperate continental climate. Mediterranean, tropical desert, and subtropical monsoon climates were the most common. Winters were frigid and summers were scorching in the temperate continental climate, characterized by significant annual and daily temperature variations and little to no rainfall during the summer months. Although these climatic conditions provided PPRV with a favorable living environment, they also posed a serious threat to the local bovine species.

The main areas of activity for Nubian ibex ( *Capra nubiana* ), Arabian gazelle ( *Gazella arabica* ), Dorcas gazelle ( *Gazella dorcas* ), four‐horned antelope ( *Tetracerus quadricornis* ), and blackbuck ( *Antilope cervicapra* ) fluctuated near the Tropic of Cancer. The most common climate types in these areas were tropical desert climate, tropical monsoon climate, and subtropical monsoon climate. There were notable regional variations as well as dry and wet differences. Tropical monsoon climate conditions predominated in the habitats of the antelope and Indian blackbuck ( *Antilope cervicapra* ). This climate was characterized by year‐round high temperatures, ample precipitation, and significant seasonal variations. Consequently, the PPRV hazard to wild animals in these regions was not as great as it was in the 30°N–50°N active range.

**FIGURE 5:**
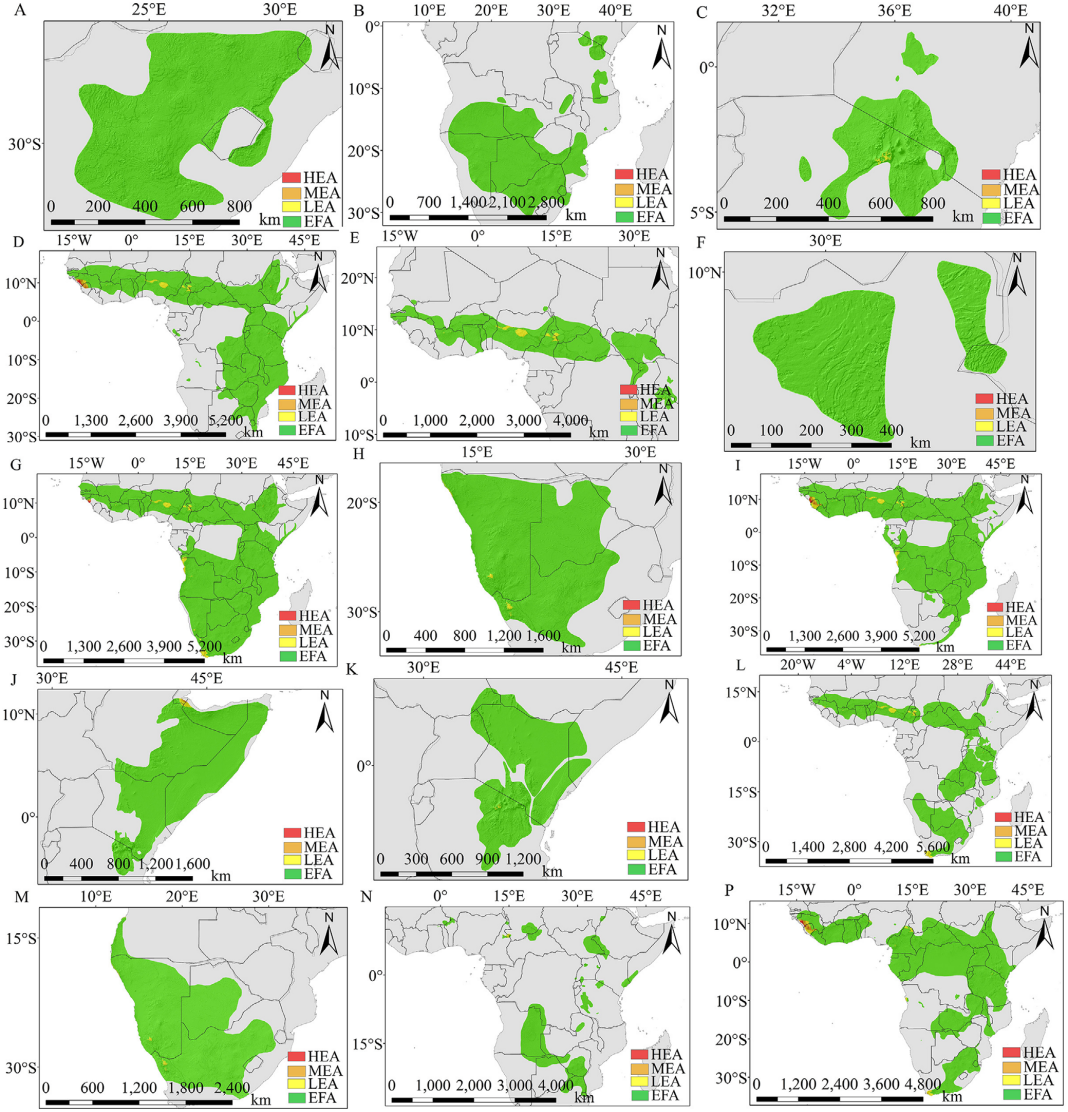
Identification and analysis of potential low‐ or no‐risk areas of peste des petits ruminants in the distribution area of wild animals of Bovidae. (A) Black Wildebeest; (B) Common Wildebeest; (C) Thomson's Gazelle; (D) Waterbuck; (E) Kob; (F) Nile Lechwe; (G) Common Duiker; (H) Gemsbok; (I) Bushbuck; (J) Gerenuk; (K) Grant's Gazelle; (L) Hartebeest; (M) Springbok; (N) Topi; (P) African Buffalo.

According to the analysis of Figures 2, 3, and 5, common wildebeest ( *Connochaetes taurinus* ), black wildebeest ( *Connochaetes gnou* ), Thomson's gazelle ( *Eudorcas thomsonii* ), waterbuck ( *Kobus ellipsiprymnus* ), kob ( *Kobus kob* ), and other species had a range of activities spanning approximately 20°N–37°S across Europe, Asia, and Africa. In these regions, there was little risk of contracting PPRV due to the predominantly tropical climate. The high temperatures, rainfall, and rich vegetation in these areas created a conducive climate for the survival of wild animals, despite the limited opportunities for PPRV transmission. As a result, cattle in these regions were at little risk of contracting PPRV.

Species such as waterbuck ( *Kobus ellipsiprymnus* ), kob ( *Kobus kob* ), common duiker ( *Sylvicapra grimmia* ), gemsbok ( *Oryx gazella* ), bushbuck ( *Tragelaphus scriptus* ), hartebeest ( *Alcelaphus buselaphus* ), and African buffalo ( *Syncerus caffer* ) predominantly inhabit the regions between 10°N and 10°S. Their active areas are characterized by hot and humid climates, along with dense vegetation. The abundant food sources and habitats available in these regions support wild animals. Between 10° and 20° north latitude, the PPRV low‐to‐medium risk region was concentrated. The probability of PPRV infection for these species in this range was negligible due to the climate and vegetation characteristics that hinder the virus's propagation.

### 3.6 Identification and Analysis of Potential Risk Areas of Peste Des Petits Ruminants in Non‐Bovine Species

**FIGURE 6:**
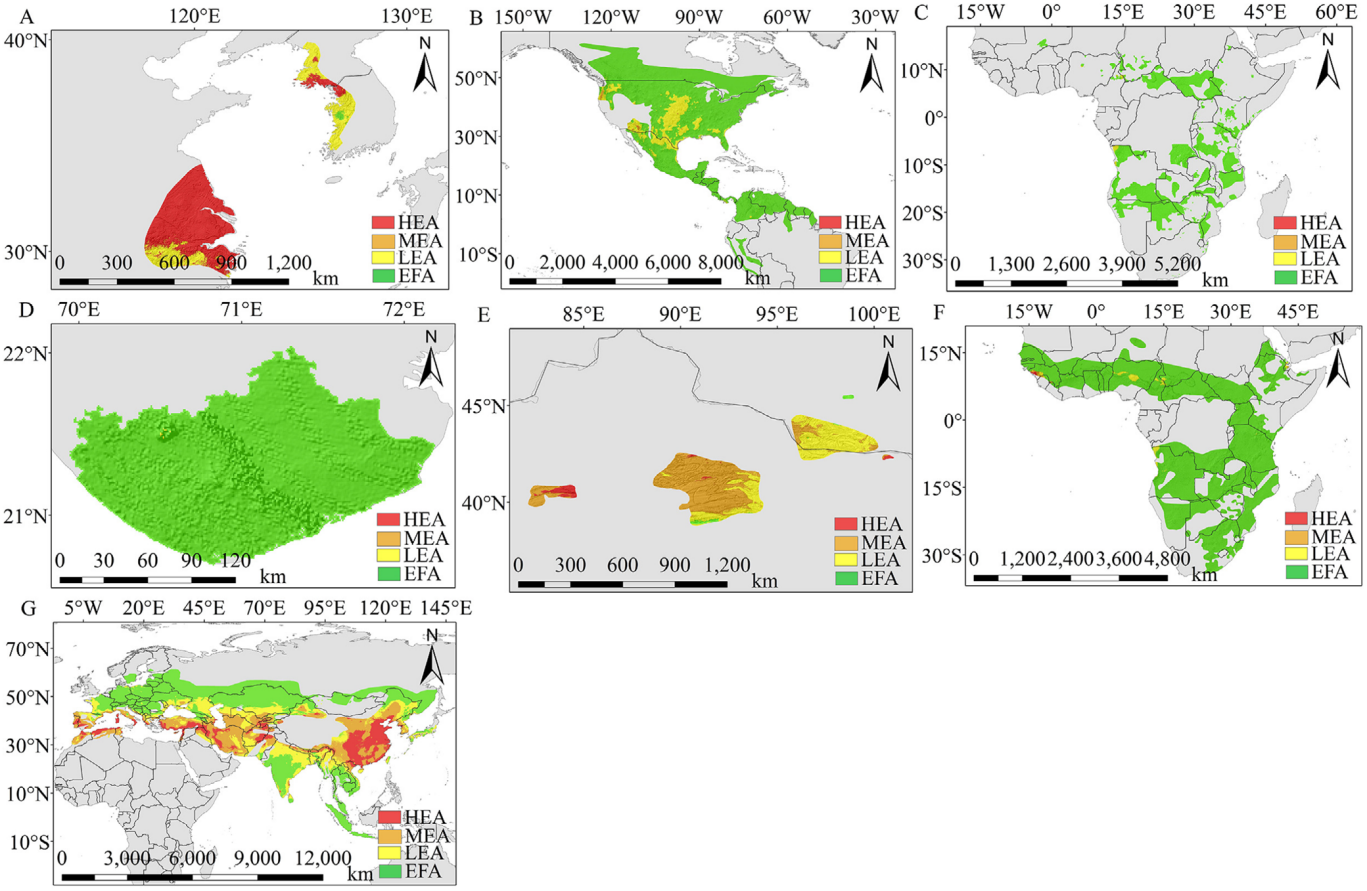
Identification and analysis of potential risk areas of peste des petits ruminants in non‐bovine species. (A) Water Deer; (B) White‐tailed Deer; (C) African Savanna Elephant; (D) Asiatic Lion; (E) Wild Camel; (F) Common Warthog; (G) Wild Boar.

White‐tailed deer ( *Odocoileus virginianus* ) and water deer ( *Hydropotes inermis* ) were primarily found in temperate and subtropical areas (Figure 6). The vegetation in these areas was varied, and the climate was moderate and humid, resulting in minimal chances of contracting PPRV. The majority of Asiatic lions ( *Panthera leo persica* ) and African savanna elephants ( *Loxodonta africana* ) inhabit tropical grasslands with conditions that limit virus transmission, despite their diverse range of activities and variable climates. In contrast, wild boars ( *Sus scrofa* ), common warthogs ( *Phacochoerus africanus* ), and wild camels ( *Camelus ferus* ) are primarily found in dry or semi‐arid regions (Figure 6). There was little chance of infection in these arid climates, as the conditions didn't support the survival of the virus. The characteristics of the species' distribution, combined with the climate conditions, contributed to the low infection risk of PPR.

The topography was varied, with these species living between 0 and 4500 m above sea level. Significant vertical climatic change occurred from low‐lying plains to alpine meadows, which further influenced the virus's transmission pathways. The climate was often dry or semi‐arid, which limited virus activity and maintained a minimal risk of infection for these species at various altitudes, even when heights varied greatly. These species also exhibited a relatively limited range of activity and weak movement behaviors, which further reduced the risk of PPRV transmission by minimizing contact with high‐risk locations. There were also species variations, and these species had differing levels of viral resistance. Certain animals possessed innate antibodies that enhanced their resistance to illness. According to several criteria, the overall risk of PPRV infection in these non‐bovine species in that environment was not significant.

## 4 Discussion

### 4.1 Application of MaxEnt Model in Infectious Diseases

In this study, we developed a high‐precision risk assessment model to elucidate the transmission of PPRV across various wildlife habitats. This model integrated the MaxEnt approach with global PPRV epidemiology data and climate environment variables. It established a robust framework for the development of future preventive and control measures, providing a scientific basis for wildlife protection. However, despite the model's high accuracy, the issue of data bias should not be overlooked (Wang et al. 2020). For example, the coverage density of data on livestock epidemics published by the Animal Husbandry and Veterinary Bureau of the Ministry of Agriculture and Rural Affairs of the People's Republic of China is substantially higher than that for wild animal data (Bureau of Animal Husbandry and Veterinary Medicine, Ministry of Agriculture and Rural Affairs of the People's Republic of China 2020). Additionally, this study encounters significant data limitations, particularly concerning the availability of high‐resolution environmental variable data. Notably, the climate data utilized does not cover the period from 2021 to 2024. This gap creates a deficiency of environmental predictors that align with species occurrence data during the testing period, which could impact the model's sensitivity and, as a result, the overall assessment of PPRV transmission risk. Similar issues have been reported in studies focused on predicting the distribution of Lyme disease vectors (Slatculescu et al. 2020). Despite the identified data biases and statistical errors, the application of the MaxEnt model offers valuable new insights for assessing the risk of PPRV. Future research should prioritize the collection of long‐term dynamic environmental data and optimization data collection methods to enhance model accuracy. Additionally, strengthening the dynamic monitoring of wildlife habitats and climate change is crucial for improving predictive capabilities related to PPRV transmission.

### 4.2 Environmental Factors and Ecological Effects of PPRV Transmission

The study's findings indicate that the two most significant variables influencing PPRV transmission are the annual mean temperature (Bio1) and temperature seasonality (standard deviation × 100) (Bio4), which together account for 52.3% of the total variance. The risk of PPRV transmission peaks when the annual average temperature reaches 17.5°C (Table 3 and Figure 1A,D), which aligns with the results of a PPRV transmission risk analysis conducted within the Russian Federation (Agoltsov et al. 2022). When the average annual temperature is approximately 10°C, the Venezuelan equine encephalitis virus (VEEV) spreads most effectively (León et al. 2020). The impact of the temperature seasonal variation coefficient (Bio4), with a peak of 880, indicates the highest risk for PPRV transmission and reflects the virus's adaptability to climate change. Additionally, a study on the risk factors for highly pathogenic avian influenza H5N6 in China found that the timing of H5N6 outbreaks varied with different diurnal temperature fluctuations across seasons (Sun et al. 2023). Temperature fluctuations primarily influence the transmission risk of the virus by modifying host immune responses and viral survival rates (Altizer et al. 2013). The impact of temperature seasonality peaks at around 880, significantly affecting viral transmission. Subsequently, as annual temperature variations increased, viral transmission was inhibited. When the seasonal temperature variation coefficient equaled or exceeded 1700, viral transmission was almost completely suppressed. Temperature changes may influence viral transmission through two pathways (Altizer et al. 2013; Uwishema et al. 2023). First, they extend the virus's survival time in the environment (Morris et al. 2021) (such as low temperatures inhibiting viral activity). Second, they modify host behavioral patterns (Gibb et al. 2020) (drought forces hosts to congregate near water sources).

The contribution of precipitation in March (Prec3) to the spread of PPRV (5.6%) was lower than the temperature factor; however, precipitation levels between 0 and 100 mm also in March had a significant impact on viral transmission. This change may alter the aggregation patterns of wild hosts by influencing vegetation growth and water distribution, thereby increasing the likelihood of virus transmission (Casades‐Martí et al. 2025). Small fluctuations in precipitation can significantly change the distribution of water sources, prompting animals to congregate around these areas and thereby increasing the risk of contact transmission (Bezirtzoglou et al. 2011). A similar phenomenon has been observed in studies of African swine fever, where resource scarcity during the dry season led to an increased frequency of contact between wild boars and domestic pigs in artificial water sources, which was a key factor in the virus's spread (Aguilar‐Vega et al. 2024).

Furthermore, based on the geographic data provided by the OIE, it has been found that countries experiencing significant outbreaks of PPRV share common characteristics (The World Organization for Animal Health (OIE) 2025). Epidemics occurring in coastal or river‐adjacent areas were predominantly found in high‐risk regions. The humid climate in these environments facilitates virus transmission, while frequent international trade and personnel mobility further exacerbate the risk of epidemic spread (The World Organization for Animal Health (OIE) 2025). In the past 5 years, Hungary, Greece, Romania, and Bulgaria—countries reporting PPRV infection—were first noted on the official website of the OIE. These countries are situated north of 30 degrees latitude (The World Organization for Animal Health (OIE) 2025), which aligns closely with our research results. These findings further underscore the impact of global climate change on the transmission patterns of infectious diseases (Baker et al. 2022).

### 4.3 Habitat Overlap and Contact Frequency

This study found that areas with a high risk of PPRV transmission—such as western Mongolia, Xinjiang in China, and Bulgaria—typically exhibit significant overlap between the habitats of wild animals and livestock. Notably, Mongolia experienced several PPR incidents in 2016 (Pruvot et al. 2020). The habitat of the Mongolian Saiga antelope significantly overlaps with the grazing areas utilized by herders, facilitating direct contact between wild animals and livestock during virus outbreaks. This pattern aligns closely with the findings of this study. Similar dynamics have been observed with other species, such as the blue sheep, which also experience heightened risks of virus exposure (Pruvot et al. 2020; Li et al. 2017). PPRV infection events at the wildlife‐livestock interface in the Greater Serengeti ecosystem (Jones et al. 2021) are consistently associated with contact between domestic animals and habitat overlap. This perspective is further supported by studies on African swine fever (Aguilar‐Vega et al. 2024). The high‐risk areas identified in this study, such as Mongolia and parts of China, are primarily agro‐pastoral ecotones. The frequent interactions between domestic and wild animals, coupled with the migration of wild animals, have exacerbated the spread of the virus.

This habitat overlap increases the likelihood of virus transmission and complicates epidemic control efforts (Zhou et al. 2023). The migratory behavior of wild animals and the ranging activities of domesticated animals create a natural bridge for the spread of viruses. Xu et al. (2025) have confirmed this perspective in their research on the genetic diversity and transmission dynamics of coronaviruses. Research has demonstrated that climate change enhances virus transmission across species by increasing habitat overlap, which consequently diversifies the transmission pathways between different species (Carlson et al. 2022). In this study, PPRV was found to infect 38 different host species, indirectly affirming the diversity of these transmission pathways. Furthermore, the expansion of human activity has further encroached upon the living spaces of wild animals, leading to more frequent interactions with domestic animals and increasing the risk of virus transmission between different ecosystems.

### 4.4 Species Differentiation

Geographical differences among significant variations in the adaptability and transmission efficiency of viruses across different hosts (Carlson et al. 2022). Within the 38 species of wild animals examined in this study, 17 species were confirmed to be susceptible to PPRV (SowjanyaKumari et al. 2021). Geographical differences contribute to a significant threat of PPRV infection among various species. The habitat overlap and frequent interactions among wild boar, wild goat, Mongolian Saiga antelope, and other species increase the risk of PPRV infection for these animals (EFSA Panel on Animal Health and Welfare et al. 2021), thereby exacerbating biodiversity loss (Carlson et al. 2022). PPRV has four subtypes, and different subtypes have different transmission capabilities in different regions. Types I and II are spread in West Africa, while type III is limited to East Africa, and all strains reported in Asia and the Caucasus belong to type IV (Courcelle et al. 2024; Banyard et al. 2010).

Species differentiation contributes to the complexity of virus transmission. Variations in physiological characteristics among species affect the transmission efficiency and adaptability of viruses in different hosts (Courcelle et al. 2024). PPRV comprises four subtypes, each exhibiting varying infection characteristics across different species (Banyard et al. 2010). Studies have indicated that wild boars may serve as a key vector for PPRV transmission due to their wide distribution and high frequency of contact with other species (Schulz et al. 2018). Among wild bovine species, both the transmission efficiency and pathogenicity of PPRV vary (Rahman et al. 2020), which is closely linked to their physiological traits and habitat conditions. Additionally, wild goats and Mongolian Saiga antelopes exhibit high susceptibility to PPRV, attributed to their unique immune system (Banyard et al. 2010). In sheep and domestic goats, the transmission pathways of PPRV are more complex, and the close contact inherent in domestic environments accelerates the speed of virus transmission. The pathogenicity of different PPRV subtypes varies in sheep and goats; studies indicate that the pathogenicity of PPRV in goats is generally higher than in sheep, which is linked to the immune system and physiological characteristics of goats (Truong et al. 2014). Species differentiation in viral transmission has also been demonstrated in studies on the H9N2 avian influenza virus (Yang et al. 2025; Kellner et al. 2025).

### 4.5 PPRV Risk Analysis Based on Suitability Model and Historical Data

The results of this study indicate that the transmission risk of PPRV gradually increases from west to east and is generally located in a strip‐shaped geographical pattern ranging from 10°N to 50°N (Figure 2). This unique spatial distribution pattern is not accidental but is jointly driven by a series of interrelated natural geographical, socio‐economic, and agricultural and pastoral activity factors (Agoltsov et al. 2022; Legnardi et al. 2022). First of all, climate and ecological factors are the natural basis for the formation of this ribbon‐like pattern. The latitude range of 10°N–50°N roughly delineates the ecological climate zone suitable for the survival of PPRV hosts (mainly small ruminants such as goats and sheep) and viruses in the external environment (Zhao et al. 2021). This region avoids overly hot and humid tropical rainforests (such as those near the equator) and overly cold and dry high‐latitude or high‐altitude areas (Agoltsov et al. 2022). The increasing trend of risk from west to east is likely related to the gradient changes in precipitation and vegetation coverage (Niu et al. 2021; Zafar et al. 2024; Zeng et al. 2021). The western part of the study area (such as parts of Central Asia and West Asia) is usually drier, with grasslands and deserts as the main vegetation, and the density of animal husbandry is relatively low. In regions like East Asia and South Asia, precipitation is gradually increasing, and the agricultural‐pastoral ecotone is more complex, providing more favorable conditions for animal aggregation and virus transmission (Zhang et al. 2022). The results of our MaxEnt suitability model are in agreement with the global ecological regionalization, confirming that climate factors are the primary constraint determining the potential distribution of PPRV. Secondly, the agricultural and pastoral system and trade activities are the key human‐driven factors leading to the “high in the east and low in the west” risk pattern (Legnardi et al. 2022). PPRV mainly affects small ruminants, and its spread is closely related to the movement and trade of live animals. The eastern regions with higher risks (such as parts of China, Bangladesh, and India, etc.) are often concentrated areas for small ruminant breeding and active areas for live livestock trade globally or regionally (Robinson et al. 2014; Kumar et al. 2014). Frequent animal movements, especially in the absence of effective supervision and vaccination, have greatly increased the chances of virus transmission (Kumar et al. 2014). In contrast, some regions in the west may be dominated by nomadic or extensive animal husbandry, with relatively low breeding density and cross‐regional trade frequency. This might to some extent limit the rapid spread of the virus. Therefore, our risk map not only reflects the ecological suitability of the virus but also superimposes the imprint of human activities. Thirdly, the high consistency between historical epidemic data and model predictions verifies the reliability of the model and highlights the prevention and control pressure in high‐risk areas. Our analysis shows that the high‐risk areas identified by the model are highly consistent with the historical hotspots of PPRV outbreaks reported by institutions such as OIE (Kumar et al. 2014). This consistency indicates that an analytical framework that combines ecological suitability (potential risks) with historical outbreak data (current manifestations) can effectively identify key areas that are continuously under threat.

## 5 Conclusion

This study found that according to the analysis of PPR epidemic data from 2007 to 2024, the driving mechanism of key climatic and environmental factors such as temperature seasonality (standard deviation × 100) (Bio4), annual mean temperature (Bio1), isothermality (Bio3), mean diurnal range (Bio2), mean monthly precipitation in March (Prec3), and the maximum temperature of warmest month (Bio5) on the transmission of PPRV. These climatic factors significantly enhanced the transmission of PPRV by affecting virus survival and host behavior. The spread of PPRV showed a specific banded geographical distribution in the world, and the geographical location is about 10°N~50°N banded range. Its distribution area is mainly concentrated in some countries and regions of the Northern Hemisphere, and the risk level gradually increases from west to east. It posed a significant potential threat to various species, including Markhor ( *Capra falconeri* ), Wild Goat ( *Capra aegagrus* ), Mountain Gazelle ( *Gazella gazella* ), and Goitered Gazelle ( *Gazella subgutturosa* ). Additionally, it threatened the water deer (*Hydropotes inermis*) and the wild camel ( *Camelus ferus* ) within the Camelidae family. Habitat overlap and species differentiation jointly affect PPRV transmission. Based on the research results, a cross‐border joint defense strategy based on the restoration of wild species habitat connectivity was proposed to provide a scientific basis for the protection of biodiversity and public health security.

## Author Contributions

**Guiping Lu:** conceptualization (equal), data curation (equal), formal analysis (equal), investigation (equal), methodology (equal), software (equal), validation (equal), visualization (equal), writing – original draft (equal), writing – review and editing (equal). **Feng Jiang:** conceptualization (equal), data curation (equal), funding acquisition (equal), methodology (equal), project administration (equal), resources (equal), writing – review and editing (equal). **Jialong Guo:** investigation (equal), methodology (equal). **Lei Si:** investigation (equal), methodology (equal). **Wenrui Jiao:** investigation (equal), methodology (equal). **Rui Zhang:** investigation (equal), methodology (equal). **Daoxin Liu:** resources (equal), supervision (equal), validation (equal), visualization (equal). **Jingjie Zhang:** resources (equal), software (equal), validation (equal), visualization (equal).

## Funding

This work was supported by Chief Scientist Program of Qinghai Province, 2024‐SF‐102. The 2023 award fund of Qinghai Provincial Key Laboratory of Animal Ecological Genomics, QHEG‐2024‐04. China Postdoctoral Science Foundation, 2024T170992, 2023M743743.

## Ethics Statement

The authors have nothing to report.

## Conflicts of Interest

The authors declare no conflicts of interest.

## Supporting information


**Figure S1:** Distribution of distances from test presences to their nearest training presences (km, log10 scale). The histogram pools results from 10 independent random splits; dashed lines denote P25/median/P75 (12.49/29.65/53.07 km). The distribution indicates substantial spatial separation between most test and training samples, suggesting a low risk of information leakage from near‐neighbor effects.
**Table S1:** Per‐split statistics of spatial separation between training and test samples: nearest‐neighbor geographic distances (km) across ten random splits.

## Data Availability

The environment variables used in the study were obtained from WorldClim (http://www.worldclim.com/), World Organization for Animal Health (OIE) (https://www.woah.org/en/home/), the ‘Veterinary Bulletin’ published by the Animal Husbandry and Veterinary Bureau of the Ministry of Agriculture and Rural Affairs of the People's Republic of China (https://www.xmsyj.moa.gov.cn), International Union for Conservation of Nature (https://iucn.org/), and Natural Earth (https://www.naturalearthdata.com/). Data sharing is not applicable to this article, as no new data were created or analyzed in this study.
