# Supplementary material for: Spatial Patterns of Global Peste Des Petits Ruminants Virus and Its Potential Risk Assessment for Various Wildlife Habitats
Source: Ecol Evol. 2025 Dec 17;15(12):e72723. doi: 10.1002/ece3.72723 (PMC12712230; doi:10.1002/ece3.72723)
Supplement: Supplementary file 1 — Figure S1: Distribution of distances from test presences to their nearest training presences (km, log10 scale). The histogram pools results from 10 independent random splits; dashed lines denote P25/median/P75 (12.49/29.65/53.07 km). The distribution indicates substantial spatial separation between most test and training samples, suggesting a low risk of information leakage from near‐neighbor effects. Table S1: Per‐split statistics of spatial separation between training and test samples: nearest‐neighbor geographic distances (km) across ten random splits. [file ECE3-15-e72723-s001.docx]

**Fig. S1.** Distribution of distances from test presences to their nearest training presences (km, log10 scale). The histogram pools results from 10 independent random splits; dashed lines denote P25/median/P75 (12.49/29.65/53.07 km). The distribution indicates substantial spatial separation between most test and training samples, suggesting a low risk of information leakage from near-neighbor effects.


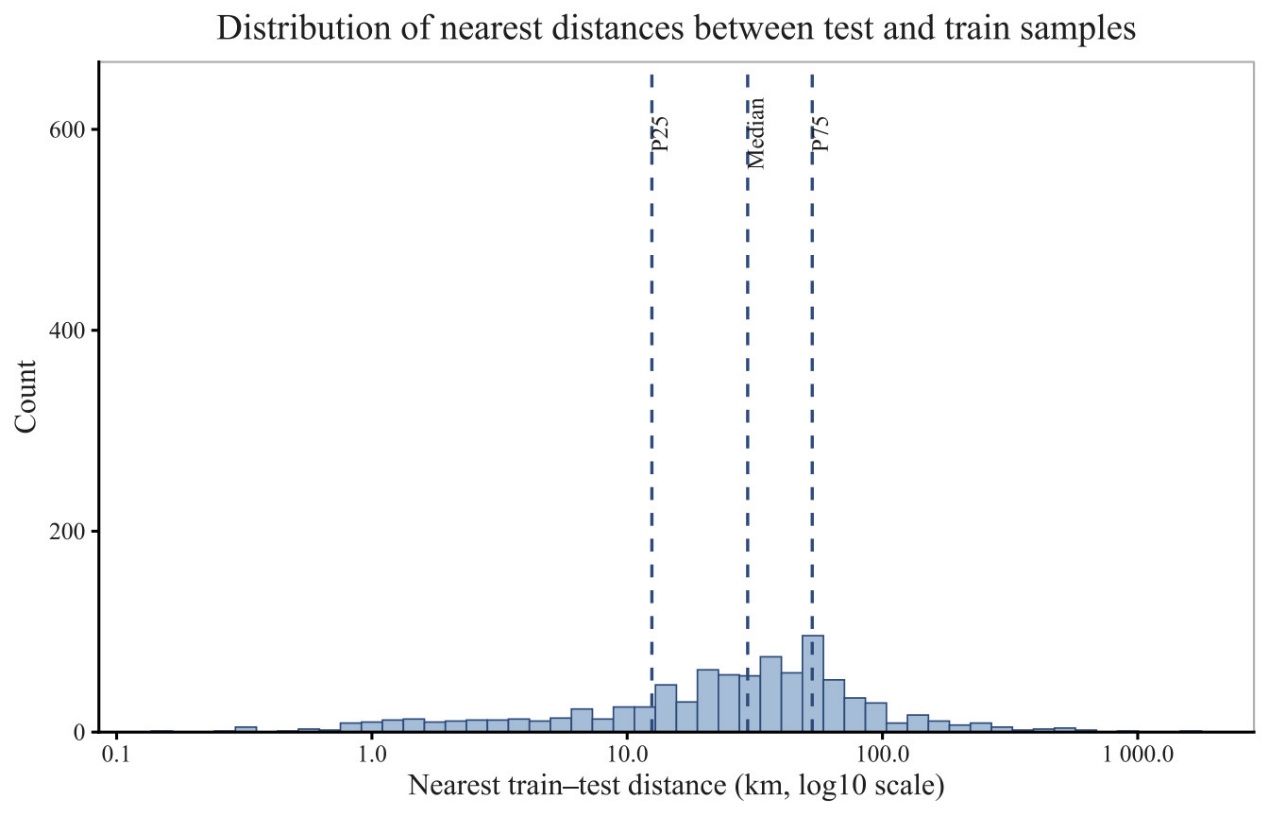


**Table S1**. Per-split statistics of spatial separation between training and test samples: nearest-neighbor geographic distances (km) across ten random splits

| split_id | n_test | min_km | p25_km | median_km | p75_km | max_km |
| --- | --- | --- | --- | --- | --- | --- |
| 1 | 90 | 0.811 | 11.688 | 27.115 | 51.035 | 532.139 |
| 2 | 90 | 0.341 | 15.553 | 36.406 | 54.569 | 1729.823 |
| 3 | 90 | 0.933 | 15.014 | 34.070 | 53.880 | 940.034 |
| 4 | 90 | 0.272 | 12.364 | 31.661 | 52.567 | 574.332 |
| 5 | 89 | 0.308 | 13.647 | 37.545 | 64.564 | 429.420 |
| 6 | 89 | 0.296 | 6.269 | 25.641 | 49.619 | 311.021 |
| 7 | 89 | 0.161 | 9.932 | 26.446 | 45.325 | 182.288 |
| 8 | 89 | 0.341 | 12.337 | 26.672 | 55.879 | 360.333 |
| 9 | 89 | 0.618 | 15.563 | 27.552 | 56.491 | 675.165 |
| 10 | 89 | 0.569 | 14.906 | 31.135 | 55.486 | 360.333 |
